# Supplementary material for: Targeted Therapy with PI3K, PARP, and WEE1 Inhibitors and Radiotherapy in HPV Positive and Negative Tonsillar Squamous Cell Carcinoma Cell Lines Reveals Synergy while Effects with APR-246 Are Limited
Source: Cancers (Basel). 2022 Dec 23;15(1):93. doi: 10.3390/cancers15010093 (PMC9818008; doi:10.3390/cancers15010093)
Supplement: Supplementary file 1 [file cancers-15-00093-s001.zip › cancers-2113207-supplementary.pdf]

cancers

MDPI

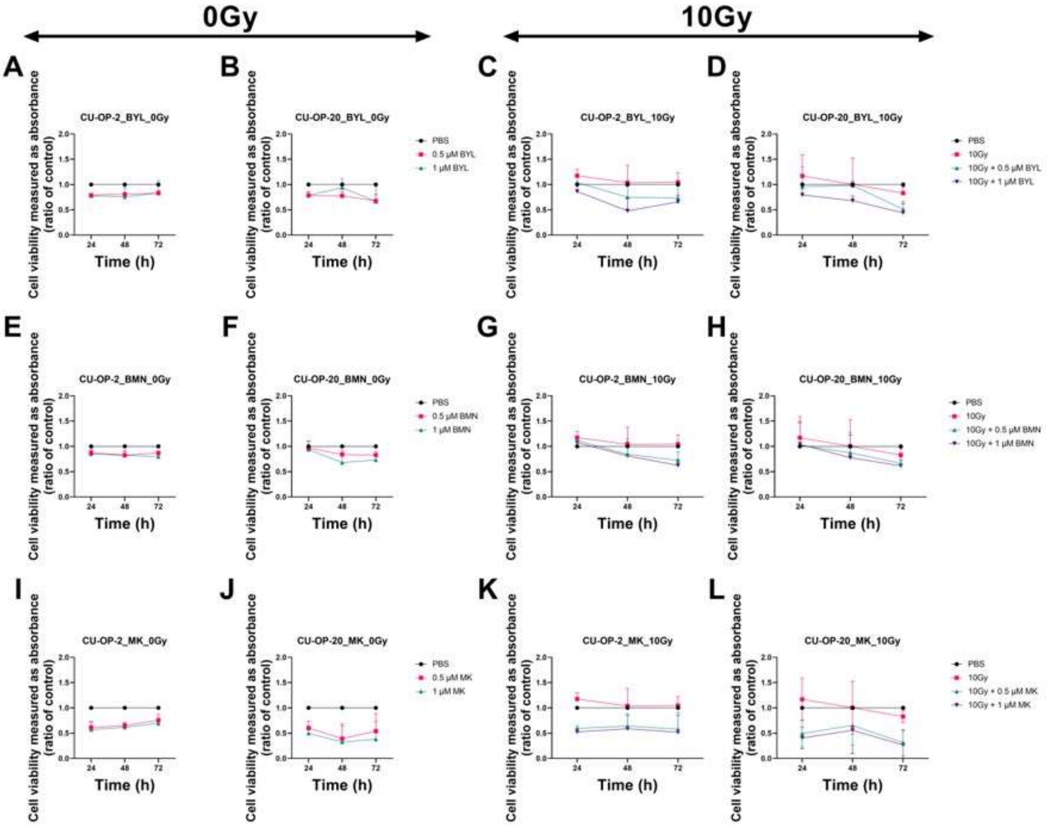

Figure S1. Viability assays on HPV+ CU-OP-2 and CU-OP-20 with single inhibitors with and without irradiation. WST-1 viability assays on HPV+ CU-OP-2 and CU-OP-20 with viability measured as absorbance 24-72 h after single treatments with 0.5-1 μM BYL719 **(A-D)**, BMN-673 **(E-H)** and MK-1775 **(I-L)**, with/without 10 Gy. BYL denotes BYL719, BMN denotes BMN-673 and MK denotes MK-1775.

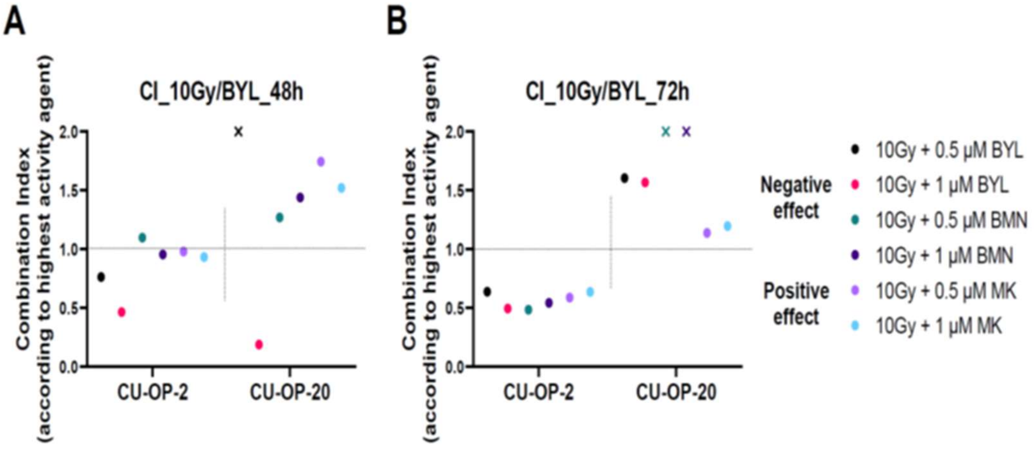

Figure S2. Combinational effects on HPV^{+} CU-OP-2 and CU-OP-20 with single inhibitors and irradiation. Combinational effects on HPV^{+} CU-OP-2 and CU-OP-20 upon adding 10 Gy to PI3K inhibitor BYL719, to PARP inhibitor BMN-673 and to WEE-1 inhibitor MK-1775 **(A-B)**. Combination indexes (CIs) were shown with the highest single agent approach after 48 h **(A)** and 72 h **(B)**, where CI< 1 shows a positive and CI> 1 a negative combination effect. CIs were calculated from the mean of three experiments analysed by WST-1 assays at 48 and 72 h after treatment. BYL denotes BYL719; and BMN denotes BMN-673 and MK denotes MK-1775.

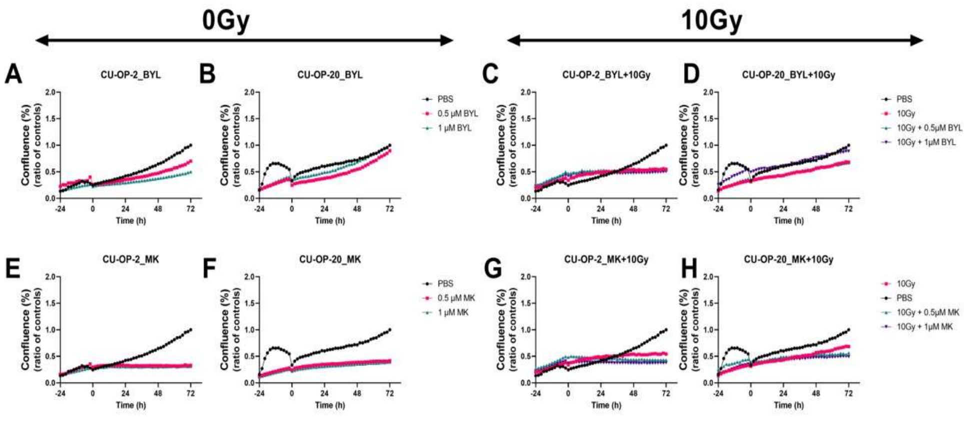

Figure S3. Proliferation responses of HPV$^+$ CU-OP-2 and , CU-OP-20 cell lines with single inhibitors and irradiation. Proliferation responses sponses of HPV$^+$ CU-OP-2, CU-OP-3, CU-OP-20, and HPV$^-$ CU-OP-17 after treatment with PI3K inhibitor BYL719 (**A**–**D**) or with WEE1 inhibitor MK-1775 (**E**–**H**) with/without 10 Gy. The graphs represent one run per cell line. Confluence (%) denotes proliferation response; BYL denotes BYL719 and MK denotes MK-1775.
